# Do different cone beam computed tomography exposure protocols influence subjective image quality prior to and after root canal treatment?

**DOI:** 10.1007/s00784-020-03524-w

**Published:** 2020-08-25

**Authors:** Andy Wai Kan Yeung, Basak Harper, Chengfei Zhang, Prasanna Neelakantan, Michael M. Bornstein

**Affiliations:** 1 Oral and Maxillofacial Radiology, Applied Oral Sciences and Community Dental Care, Faculty of Dentistry, The University of Hong Kong, Hong Kong SAR, China; 2 Endodontology, Faculty of Dentistry, The University of Hong Kong, Hong Kong SAR, China; 3 Department of Oral Health & Medicine, University Center for Dental Medicine Basel UZB, University of Basel, Basel, Switzerland

**Keywords:** Cone beam computed tomography, Dose optimization, Root canal treatment, Image quality, Low dose

## Abstract

**Objectives:**

The current study aimed to evaluate different CBCT exposure protocols and influencing factors affecting the subjective image quality of scans taken for endodontic indications.

**Materials and methods:**

Twelve extracted teeth, comprising of two sets of maxillary molars, premolars, canines and incisors, mandibular premolars, and molars, were endodontically treated, and either received a fiber or metal post. The teeth were scanned by CBCT imaging before and after root canal treatment, and after post insertion. Each scan was performed thrice, using an ultra low dose (ULD), standard (SM), and high-resolution mode (HR), respectively. Twelve observers—4 endodontists, 4 periodontists, and 4 radiologists—assessed the subjective image quality using visual analogue scales (VAS). Potential influencing factors were evaluated including acquisition mode, observer specialty, stage of treatment, type of post, and type of tooth, using one-way ANOVA and *T* test.

**Results:**

Teeth scanned with the ULD had the highest average VAS score (72.5), followed by HR (70.2), and SM (69.0) for values pooled from all teeth and observers. CBCT acquisition mode was not a significant influencing factor on the VAS scores. Observer specialty, stage of treatment, type of post, and type of tooth were significant influencing factors.

**Conclusions:**

Based on the present in vitro data, a low-dose CBCT mode seems not to negatively affect the perception of image quality.

**Clinical relevance:**

The findings from this in vitro study demonstrate that a low-dose CBCT mode might have potential for diagnostics prior to or following endodontic treatment.

## Introduction

The pitfalls of two-dimensional (2D) radiographic imaging in endodontics are well known [1]. For diagnostic purposes, conventional radiographs may have limitations, such as to identify all root canals including blocked canals, evaluate the morphology of tooth and root canal systems, localize broken instruments, and assess the relationship between the roots and the maxillary sinus or the mandibular canal [2]. Detection of cracks/fractures is also challenging using conventional radiographs, when the fracture lines are along the direction of the radiation beam [3]. If available, a surgical microscope can be helpful [4]. Three-dimensional (3D) imaging techniques such as cone beam computed tomography (CBCT) can help offset many of these problems. Nevertheless, there is considerable ambiguity in offering a decisive suggestion to routinely use CBCT for diagnostic procedures in endodontics. This is mainly related to the higher radiation dose of CBCT relative to conventional radiography [5, 6].

Contemporary root canal treatment demonstrates a steady shift toward conservation of tooth structure, and minimally invasive access cavity designs are being increasingly suggested [7]. With less direct intraoral visualization, the relevance of radiographic diagnostic imaging to offer more information regarding the root canal anatomy of a tooth prior to root canal treatment is becoming more important especially if a surgical microscope is not available [8]. However, the radiation dose of CBCT, despite being lower than that of conventional CT, negates routine clinical usage, when one applies the principles of as low as reasonable achievable (ALARA) and as low as diagnostically acceptable (ALADA) [9]. Therefore, CBCT is still considered as an adjunctive imaging modality for endodontic purposes [5, 6, 10].

CBCT devices usually allow clinicians to scan with different protocols, such as high resolution, standard, and even low-dose modes [11]. To achieve dose optimization, clinicians should consider using low-dose protocols for CBCT scans when possible [12]. For instance, an established low-dose protocol for pediatric CBCT may reduce as much as 50% of radiation dose compared to the standard exposure as recommended by the manufacturer [13]. CBCT is recommended as an efficient method of studying root canal systems [14], and high-resolution settings have been recommended by the literature and manufacturers for assessment of root canal anatomy [15]. Thus, there is a need to investigate standard and low-dose protocols, based on the fact that the three imaging protocols—high resolution, standard, and low dose—have not yet been assessed and compared for their impact on subjective image quality to evaluate root canal systems prior to or after root canal treatment.

Therefore, the goal of the present study was to evaluate different CBCT exposure protocols and potential influencing factors on the subjective image quality of CBCT scans taken for endodontic indications. The study was designed as an in vitro investigation using a cohort of mandibular and maxillary teeth that were scanned by CBCT before root canal treatment, after root canal treatment, and after post placement. The scans used one standard, one high-resolution, and one custom low-dose protocol, respectively. The primary aim was to evaluate, if observers gave significantly different ratings to images acquired by different CBCT settings. Secondary aims included the assessment of the influence of observer specialty, treatment stage, type of post, and tooth type on the ratings for subjective image quality.

## Materials and methods

### Tooth selection

**Fig. 1:**
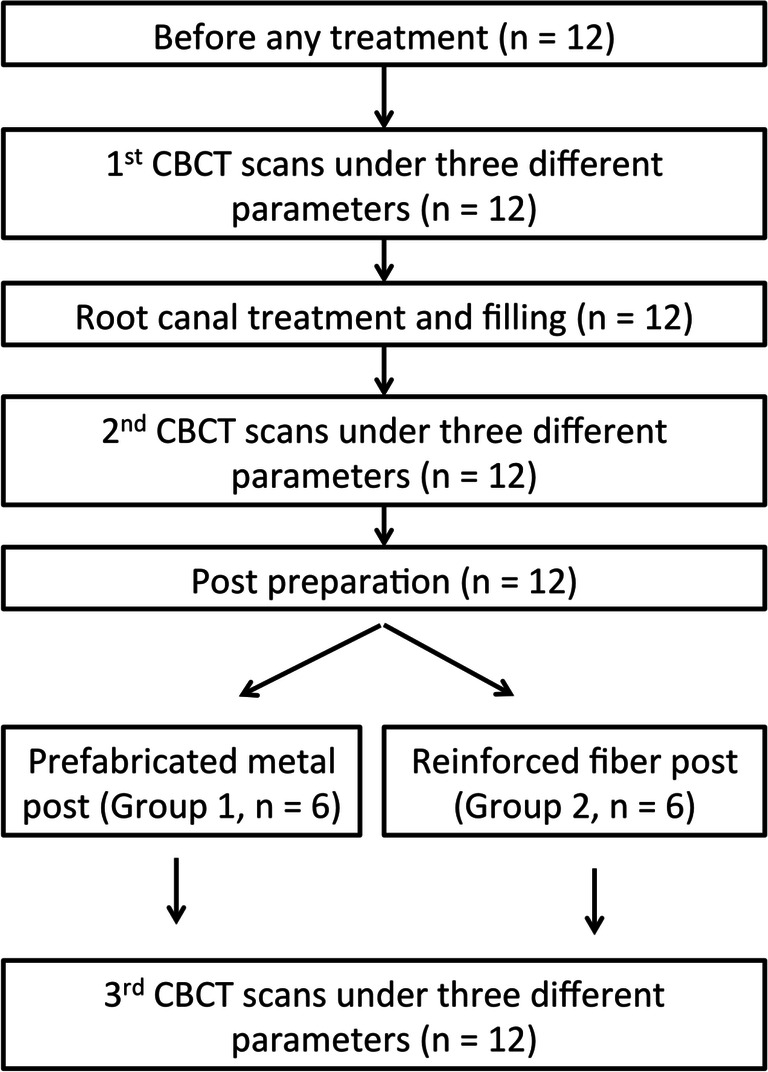
Flow-chart illustrating the workflow for the 12 teeth processed and analyzed

All extracted teeth used in this study were collected from an existing pool of teeth. The study protocol was approved by the Institutional Review Board of the university (UW 17-206). Single-rooted and multi-rooted teeth that were included for this study were divided into two groups (Fig. 1). Each group comprised of six teeth including one for each of the following: maxillary incisor, maxillary canine, maxillary premolar, maxillary molar, mandibular premolar, and mandibular molar. Premolars and molars from the maxilla and mandible were included due to their heterogenous root canal morphology. The first group of teeth received root canal treatment with a fiber-reinforced composite post, whereas the second group of teeth received root canal treatment with a prefabricated metal post. Thus, for the two study groups tested (i.e., fiber-reinforced composite post against metal post), this resulted in a total of 12 teeth to be included.

### Pre-operative CBCT scanning

**Fig. 2:**
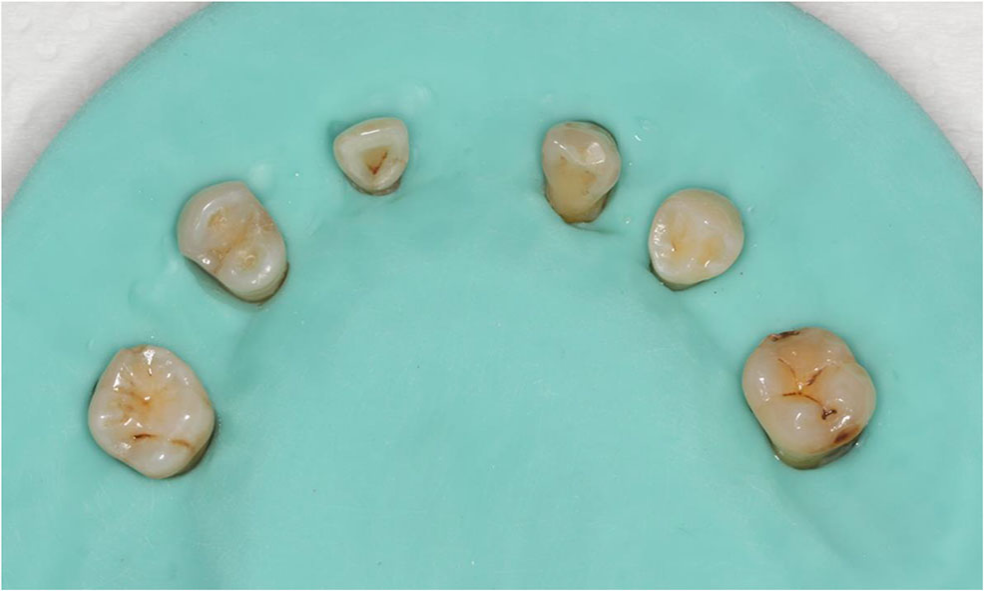
Representative image of the six teeth from one group mounted in a customized silicone mold simulating soft tissues prior to root canal treatment

According to the method described by Shelley et al. [16], six teeth from each group were mounted in a customized silicone mold simulating soft tissues (Fig. 2) for CBCT scans with the ProMax 3D Mid device (Planmeca Oy, Helsinki, Finland) using three different protocols:

1. High-resolution mode (HR): 90 kV, 10 mA, exposure time 15 s, field of view (FOV) of 8 × 5 cm, voxel size of 0.15 mm
2. Standard mode (SM; default setting given by the manufacturer): 90 kV, 8 mA, exposure time 12 s, FOV of 8 × 5 cm, voxel size of 0.2 mm
3. Ultra low dose mode (ULD; as provided by the manufacturer): 90 kV, 5.6 mA, exposure time 4 s, FOV of 8 × 5 cm, voxel size of 0.2 mm

### Root canal treatment

Root canals of all 12 teeth were prepared with Wave One Gold instruments “Medium” size (Dentsply Sirona Endodontics, York, PA, USA) using 5 mL of 2% sodium hypochlorite as the root canal irrigant. Following completion of root canal instrumentation, the root canals were flushed with sterile saline and dried with absorbent paper points (Dentsply Sirona Endodontics, York, PA, USA). The root canals were filled using gutta-percha cones and accessory cones, with an epoxy resin-based root canal sealer (AH Plus, Dentsply DeTrey, Konstanz, Germany) using cold lateral compaction technique.

Following root canal treatment, all teeth were scanned using CBCT as mentioned previously, using 3 protocols.

### Endodontic post placement

For the prefabricated metal post (group 1), the root canal fillings of the teeth were removed after root canal treatment to leave behind a 5 mm plug of apical gutta-percha. Following this, red (1.25 mm), black (1.5 mm), and yellow (1 mm) stainless steel posts (ParaPost® XP™ Stainless Steel Post, Coltene/ Whaledent Inc., Cuyahoga Falls, OH, USA) were cemented in the root canals of anteriors, premolars, and molars respectively, using resin cement (RelyX™ Unicem cement, 3M ESPE, St. Paul, MN, USA). The teeth were then filled with composite resin material (Filtex Z100, 3M ESPE, St. Paul, MN, USA).

For the fiber-reinforced composite post group (group 2), the root canal fillings of the teeth were removed after root canal treatment to leave behind a 5 mm plug of apical gutta-percha. Afterwards, 1.6 mm (molars) and 1.9 mm (anteriors and premolars) fiber posts (RelyX™ Fiber Post, 3M ESPE, St. Paul, MN, USA) were luted with resin cement (RelyX™ Unicem cement). The teeth were then filled with composite resin material (Filtex Z100).

Following placement of the endodontic posts, all teeth were scanned using CBCT as mentioned previously, using 3 protocols.

### Image quality evaluation of the CBCT scans

The CBCT images were evaluated by 12 observers form different specialties (4 from endodontology, 4 from periodontology, and 4 radiologists; all had at least 3 years of postgraduate training that involved CBCT analysis). All observers had a total of 18 CBCT sets to analyze from 2 groups of teeth using 3 different CBCT settings (HR, SM, and ULD) at 3 different treatment stages (before root canal treatment, after root canal treatment, and after post insertion). The observers graded all scans on a visual analogue scale (VAS, 0-100) for each tooth separately assessing the subjective image quality. The observers were all instructed to focus on the sufficiency of image quality for various endodontic diagnoses, such as the evaluation of root canal filling and placement of the posts. The scans were presented to the observers unlabeled, and in a randomized sequence. The observers could modify the scans including brightness and contrast. They all evaluated the images on the same computer monitor (22-inch LED, 1920 × 1080 pixels, model 223V, Philips) in a darkened room.

### Statistical analysis

Descriptive statistics were evaluated first. Inter-observer reliability was assessed using intra-class correlation coefficients (ICC) [17]. Then, the VAS scores were compared to reveal if images acquired by ULD, SM, and HR modes had any differences in the ratings of subjective image quality. Afterwards, it was assessed if observer specialty, stage of treatment, type of post, and type of tooth were influencing factors on the VAS scores. All these factors, except type of post, were tested by one-way ANOVA. The effect of the type of post on VAS scores was tested by independent *t* test. Bonferroni correction was considered to adjust for multiple testing.

The significance level was set at *p* = 0.01. All analyses were performed in SPSS (Version 25.0, IBM Corp., Armonk, New York, USA).

## Results

**Fig. 3:**
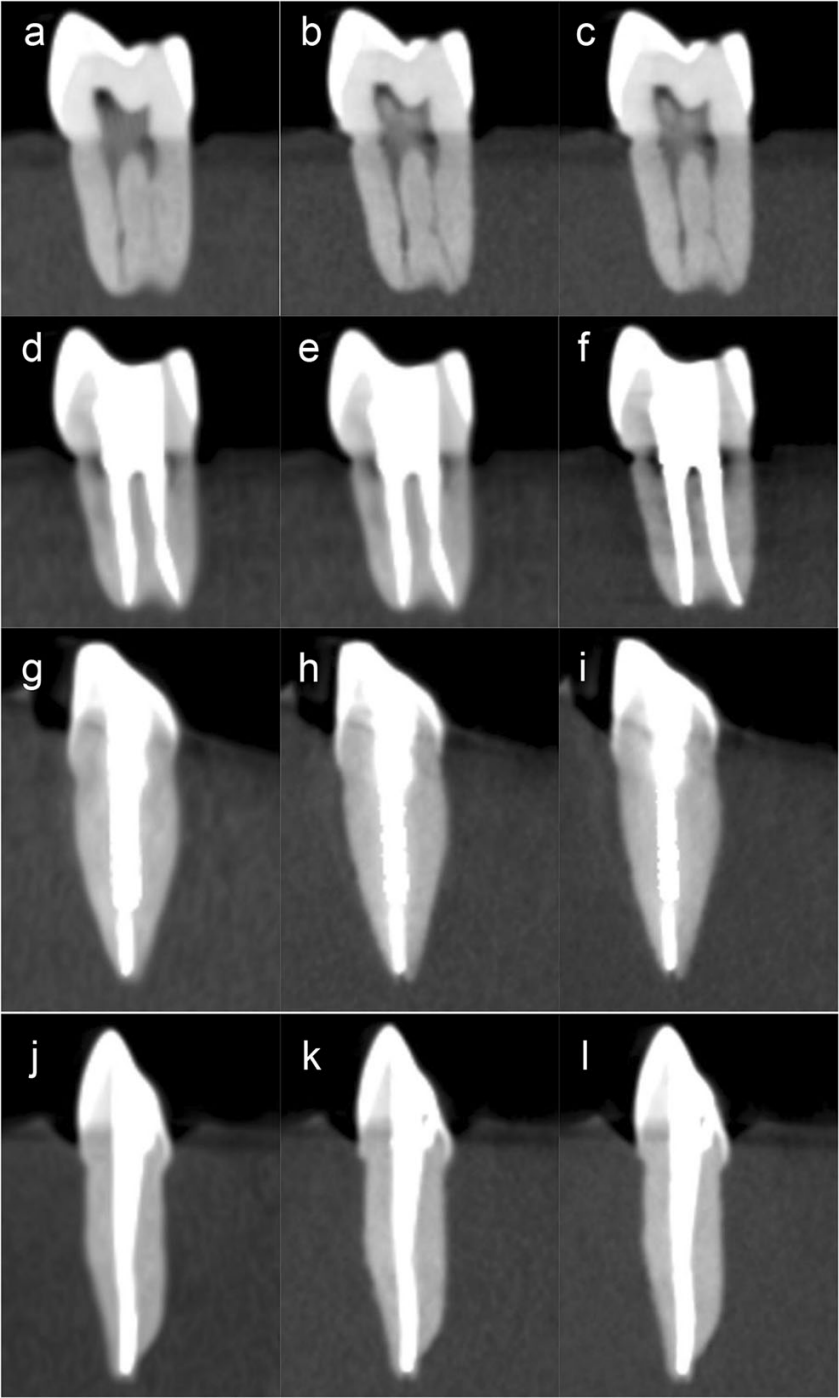
Representative CBCT images of a mandibular premolar before root canal treatment with **a** ultra low dose mode (ULD), **b** standard mode (SM), and **c** high-resolution mode (HR), and after root canal treatment with **d** ULD, **e** SM, and **f** HR. Furthermore, a maxillary canine is depicted with an inserted metal post using **g** ULD, **h** SM, and **i** HR, and with a fiber post using **j** ULD, **k** SM, and **l** HR

Representative CBCT images taken with the three exposure settings before root canal treatment, after root canal treatment, with metal post inserted**,** and with fiber post inserted are presented in Fig. 3.

### VAS scores for the three CBCT settings

Overall, teeth scanned with ULD had the highest average VAS score (72.5), followed by HR (70.2), and then SM mode (69.0) for values pooled from all teeth and all observers (Table 1). Periodontists gave the highest average VAS score (72.7, SD 17.6), followed by radiologists (70.8, SD 15.7), and endodontists (68.2, SD 20.4). With regards to the sequence of treatment, teeth scanned before root canal treatment had the highest rating (82.4, SD 11.8) compared to after root canal treatment (61.7, SD 17.4), and after post insertion (67.6, SD 17.6). Teeth with a fiber post (73.4, SD 14.8) exhibited higher ratings than those with a metal post (61.7, SD 18.5). In terms of type of tooth, maxillary canines had the highest rating (75.8, SD 17.5), followed by maxillary incisors (74.8, SD 17.9), and the molars (maxilla: 66.7, SD 23.0; mandible: 64.0, SD 24.6; Table 2).

**Table 1:** Overview of the subjective image quality of the CBCT scans for each type of tooth for all three imaging modalities from all observers (VAS scores)

	Maxilla				Mandible		Average
	Incisor	Canine	Premolar	Molar	Premolar	Molar	
Overall							
Ultra low dose mode	76.7 (15.8)	77.8 (14.9)	72.1 (19.3)	71.3 (18.3)	72.1 (19.4)	65.2 (23.5)	72.5 (15.6)
Standard mode	73.9 (18.6)	72.4 (20.5)	69.6 (21.5)	62.2 (25.1)	72.4 (19.6)	63.8 (25.1)	69.0 (18.8)
High-resolution mode	73.8 (19.3)	77.2 (16.5)	68.6 (24.2)	66.7 (24.5)	71.7 (20.2)	63.0 (25.4)	70.2 (19.5)
Before root canal treatment							
Ultra low dose mode	79.0 (14.7)	80.4 (14.6)	78.0 (16.2)	76.9 (15.8)	78.6 (14.2)	75.0 (20.1)	78.0 (13.5)
Standard mode	85.7 (9.4)	85.5 (8.2)	84.9 (10.0)	83.2 (10.2)	85.5 (8.8)	84.1 (9.5)	84.8 (8.6)
High-resolution mode	85.5 (12.8)	85.7 (12.2)	85.8 (11.6)	84.0 (13.4)	84.1 (12.8)	82.1 (11.8)	84.5 (11.9)
After root canal treatment							
Ultra low dose mode	72.0 (17.3)	73.8 (15.9)	65.3 (20.0)	66.3 (17.8)	62.1 (20.9)	57.6 (22.4)	66.2 (15.3)
Standard mode	67.5 (17.7)	67.6 (17.6)	61.1 (20.5)	52.3 (23.6)	60.1 (20.2)	52.9 (24.0)	60.2 (16.9)
High-resolution mode	65.5 (18.7)	67.5 (17.7)	54.5 (23.2)	54.2 (25.2)	59.0 (22.1)	51.6 (25.0)	58.7 (19.6)
After metal post							
Ultra low dose mode	78.6 (14.8)	78.2 (13.9)	72.8 (19.7)	68.7 (22.1)	74.4 (20.5)	63.2 (25.5)	72.6 (18.6)
Standard mode	62.4 (16.2)	54.8 (23.8)	50.3 (25.7)	42.2 (20.2)	60.8 (19.8)	43.2 (23.5)	52.3 (15.5)
High-resolution mode	68.8 (14.6)	73.9 (15.4)	58.1 (25.3)	50.3 (23.9)	65.5 (18.2)	46.1 (25.5)	60.4 (16.5)
After fiber post							
Ultra low dose mode	79.4 (15.6)	80.4 (14.4)	73.0 (21.3)	72.4 (18.4)	77.0 (17.9)	63.0 (25.7)	74.2 (14.3)
Standard mode	74.4 (24.4)	73.5 (24.2)	75.5 (11.5)	59.9 (25.4)	82.3 (10.1)	65.9 (22.2)	71.9 (16.2)
High-resolution mode	72.3 (25.4)	82.8 (12.2)	72.8 (22.7)	73.8 (16.1)	78.6 (12.7)	64.3 (24.1)	74.1 (14.9)

The VAS score ranged 0–100, with 100 being “perfect” for diagnostic purposes

SD values are in brackets

**Table 2:** Overview of subjective image quality of the CBCT scans for each type of tooth for all three specialties of observers (VAS scores)

	Maxilla				Mandible		Average
	Incisor	Canine	Premolar	Molar	Premolar	Molar	
Endo							
Before root canal treatment	90.4 (6.3)	90.6 (6.2)	88.3 (8.8)	85.5 (10.8)	88.3 (8.5)	83.5 (18.7)	87.7 (7.0)
After root canal treatment	67.0 (22.8)	68.3 (21.7)	54.4 (26.9)	51.0 (28.1)	61.0 (23.7)	52.0 (28.7)	59.0 (19.6)
After metal post insertion	66.8 (18.1)	61.9 (23.6)	53.5 (28.7)	39.0 (24.1)	60.3 (21.5)	34.3 (25.5)	52.6 (15.1)
After fiber post insertion	64.1 (31.9)	72.2 (26.1)	61.3 (25.2)	58.8 (26.6)	78.3 (14.2)	45.3 (27.0)	63.3 (15.7)
Perio							
Before root canal treatment	82.0 (13.2)	82.4 (12.3)	82.1 (12.0)	82.5 (10.1)	80.5 (14.6)	80.6 (12.6)	81.7 (12.0)
After root canal treatment	68.5 (17.0)	69.8 (15.8)	64.4 (17.7)	62.5 (20.5)	61.9 (20.9)	59.3 (20.1)	64.4 (17.7)
After metal post insertion	70.4 (18.5)	70.5 (23.5)	61.3 (26.2)	57.8 (24.2)	67.3 (22.4)	60.7 (24.2)	64.7 (22.1)
After fiber post insertion	81.3 (9.0)	83.6 (7.8)	81.7 (9.0)	73.8 (19.9)	80.1 (10.5)	75.7 (12.6)	79.3 (10.4)
Radiology							
Before root canal treatment	77.8 (14.0)	78.6 (13.4)	78.3 (16.2)	76.0 (17.3)	79.5 (11.6)	77.1 (12.2)	77.9 (13.6)
After root canal treatment	69.5 (13.1)	70.7 (13.4)	62.2 (18.1)	59.3 (18.7)	58.3 (18.3)	50.8 (21.3)	61.8 (14.9)
After metal post insertion	72.7 (12.1)	74.5 (11.7)	66.3 (19.3)	64.3 (18.0)	73.0 (14.0)	57.5 (19.3)	68.1 (14.9)
After fiber post insertion	80.8 (14.3)	80.9 (13.1)	78.3 (11.4)	73.6 (10.3)	79.6 (17.0)	72.2 (16.0)	77.6 (13.0)

The VAS score ranged 0–100, with 100 being “perfect” for diagnostic purposes

SD values are in brackets

### Inter-observer reliability

ICC values showing inter-observer reliability are listed in Table 3. Overall, the values ranged from poor to good. Endodontists and radiologists tended to have higher ICC values than periodontists. The number of ICC values (out of a total of 15) demonstrating moderate to good agreement (> 0.50) for endodontist, radiologist, and periodontist groups were 9, 7, and 5 respectively.

**Table 3:** Inter-observer reliability of VAS scores amongst different groups of specialty with different acquisition modes at each stage of treatment

	Overall	Endo	Perio	Radiology
Overall				
Ultra low dose mode	0.607	0.583	0.151	0.237
Standard mode	0.870	0.698	0.572	0.795
High-resolution mode	0.837	0.698	0.564	0.674
Before root canal treatment				
Ultra low dose mode	0.063	0.216	0.222	0.339
Standard mode	0.037	0.224	0.143	0.348
High-resolution mode	0.037	0.127	0.074	0.563
After root canal treatment				
Ultra low dose mode	0.663	0.574	0.173	0.492
Standard mode	0.549	0.284	0.119	0.436
High-resolution mode	0.428	0.107	0.274	0.063
After metal post				
Ultra low dose mode	0.745	0.680	0.098	0.581
Standard mode	0.899	0.743	0.561	0.866
High-resolution mode	0.888	0.753	0.524	0.871
After fiber post				
Ultra low dose mode	0.316	0.299	0.187	0.181
Standard mode	0.775	0.619	0.487	0.639
High-resolution mode	0.751	0.640	0.567	0.394

Intraclass correlation coefficient (ICC)

< 0.50 = poor, 0.50–0.75 = moderate, 0.75–0.90 = good, > 0.90 = excellent [17]

### Significance of potential influencing factors on VAS scores

Statistical analyses demonstrated that the CBCT acquisition mode was not a significant influencing factor on the VAS scores (*p* = 0.048) for subjective image quality (Table 4). Meanwhile, all other factors evaluated were significant influencing factors. Regarding observer specialty, periodontists gave generally higher mean VAS scores (on average 4.5 points higher) than endodontists (*p* = 0.008). Regarding stage of treatment, mean VAS score for teeth before root canal treatment was 14.8 points higher than after post insertion, which was in turn 5.9 points higher than after root canal treatment (*p* = 0.001). Teeth with fiber posts inserted had a mean VAS score that was 5.0 points higher than teeth with metal posts inserted (*p* < 0.001). With regard to the type of tooth, mean VAS scores of maxillary canines and maxillary incisors were significantly higher than those of maxillary premolars and mandibular premolars, which in turn were significantly higher than those of maxillary molars and mandibular molars (*p* < 0.001).

**Table 4:** Analysis of potential influencing factors on the subjective image quality of CBCT images

Influencing factor		Statistical test	*P* value
Acquisition mode	(1) ULD^a^	One-way ANOVA	0.048
	(2) SM^a^		
	(3) HR^a^		
Observer specialty	(1) Endodontology^a^	One-way ANOVA	0.008
	(2) Radiology^a,b^		
	(3) Periodontology^b^		
Stage of treatment	(1) Before root canal treatment^a^	One-way ANOVA	0.001
	(2) After root canal treatment^b^		
	(3) After post insertion^c^		
Type of post	(1) Metal^a^	*T* test	< 0.001
	(2) Fiber^b^		
Type of tooth	(1) Upper molar^a^	One-way ANOVA	< 0.001
	(2) Upper premolar^b^		
	(3) Upper canine^c^		
	(4) Upper incisor^c^		
	(5) Lower premolar^b^		
	(6) Lower molar^a^		

Groups identified by different superscripts were significantly different at *p* < 0.01^*^

^*^ Bonferroni correction = significance level/the number of hypotheses (0.05/5)

## Discussion

The primary aim of the current study was to evaluate, if observers gave significantly different ratings to CBCT image quality acquired by different exposure settings. Secondary aims were to assess the influence of observer specialty, treatment stage, type of post, and tooth type on VAS ratings. The major findings were that CBCT images acquired by different settings did not result in significantly different ratings. On the contrary, it was found that rather the other factors like observer specialty, treatment stage, type of post, and tooth type were influencing the VAS values, and thus, the subjective perception of image quality significantly.

The present findings are relatively novel, as high-resolution CBCT images have been often recommended and reported to be superior to low-dose and standard modes for endodontic indications, such as to assess root fractures with higher accuracy [18, 19]. However, the use of high-resolution CBCT imaging has been reported to result in higher radiation doses specifically to the eye lens and thyroid gland [20]. Indeed, the use of low-resolution CBCT imaging has been recommended for evaluating endodontic surgery with retrograde root filling [21]. However, that recommendation was based on the calculation of contrast-to-noise (CNR) ratio that involved no clinical judgment. The present evaluation was based on subjective perception of the image quality by different specialists in dental medicine, whereas previous studies either focused on observers’ accuracy on detecting lesions on CBCT images, or the more mechanistic CNR value that involves no direct observer judgment. Regarding the assessment of root canal morphology, previous studies have demonstrated the usefulness of CBCT imaging for this evaluation in both deciduous and permanent teeth, without investigating into the optimal CBCT setting for this purpose [14, 22]. Results from the present study imply that CBCT scans with a low-dose protocol may be adequate for evaluation of root canal systems.

There are no reports on the effect of different observer specialties on the subjective image quality perception of CBCT images. A recent study reported that periodontists are more likely to change their treatment plan and suggest extraction of teeth when CBCT images were provided to them to assess moderate to difficult endodontic cases, a finding that was contrasting outcomes from endodontists [23]. For non-CBCT images, it has been shown that endodontists demonstrated better inter-observer agreement on diagnosing periapical lesions with periapical radiographs [24]. Meanwhile, observer specialty was not a significant influencing factor on the resulting subjective image quality assessment when pathologists, oral medicine specialists, radiologists, and oral surgeons used panoramic images to diagnose oral unilocular radiolucent lesions [25]. Nevertheless, the results of the present study suggest that endodontists may be more consistent in radiographic assessments for evaluation of root canal systems prior to and following treatment using 3D imaging techniques. Furthermore, as periodontists gave generally higher mean VAS scores in the present study, it might be suggested that radiologists and endodontists seem to be more critical concerning CBCT image quality for the evaluation of endodontic indications.

The observer ratings for CBCT images of the teeth after fiber post insertion were generally higher than those after root canal treatment prior to post insertion. These findings are not totally clear, but it may be speculated that the observers tended to focus on evaluating the root filling materials, such as their homogeneity and length, when they rated the images without posts inserted. Thus, they may have also assessed somewhat the quality of the root canal filling itself rather than the actual image quality. A recent ex vivo study reported that root-filled teeth resulted in CBCT images of inferior quality to detect apical periodontitis compared to non-root-filled counterparts [26]. CBCT imaging has also been said to be inferior to periapical images produced by F-speed films and phosphor plates to evaluate homogeneity and length of root fillings [27]. For posts, especially metallic ones, inserted after conventional root canal treatment, the impact of scatter and noise on the subjective image quality has to be taken into serious account [28], which makes it even more important to select the imaging modality with the best potential, but lowest radiation dose exposure. In the current study, teeth after metal post insertion, but not fiber post, had the lowest mean VAS scores, compared to those before root canal treatment and after root canal treatment without post. This clearly shows that the presence of a metal post will result in scatter and noise that is detrimental for the subjective image quality of CBCT scans. Moreover, the scatter and blooming caused by endodontic filling materials may mask cracks, narrow canals, and resorption lesions that are present on the treated or the adjacent teeth in clinical reality.

The type of tooth was a significant influencing factor on VAS score. This could be related to the number of root canals in the respective teeth. Anterior teeth contain fewer canals than premolars, which subsequently contain fewer canals than molars [29]. It was reasonable for observers to give a lower score for CBCT images that exhibit a rather complex root canal system with multiple canals, relative to a system with only one or two canals.

A recent survey has revealed that half of the endodontists in the USA had a CBCT device on-site [30]. Among the various indications, more than 50% of endodontists used CBCT imaging (“occasionally,” “frequently,” or “always”) to detect missing canals. A similar survey from the UK also pointed out that assessment of complex root canal systems was one of the most important indications for the use of CBCT [31]. For both countries, CBCT images prescribed by endodontists most frequently involved small FOVs [30, 31]. These findings highlight the importance of the current evaluation on the effects of different exposure protocols (ULD, SD, and HR) on the image quality of CBCT images with small FOVs.

A relevant limitation of the current study was its in vitro setting. The teeth specimens were scanned without scatter and absorption from bone or skull materials or metallic crowns on adjacent teeth which might have influenced reconstruction and artefact expression, and no motion artifact was involved. The soft tissue simulated by silicone looked similar to the reported tissue simulation with water, which also focused on the subjective image quality of CBCT images [32]. Therefore, the results were obtained under relatively ideal circumstances, and will most likely result in less favorable ratings in a clinical scenario. These findings have to be interpreted with some caution, and need to be tested and validated under clinical conditions. Besides, the study only tested the low-dose protocol from one single CBCT device, as the present investigation was focused on the effect of specialists’ different backgrounds rather than the differences between various CBCT devices. Moreover, there are certainly alternative methodologies to assess and compare subjective image quality of CBCT scans, such as the recognition or linear measurement of detectable anatomical structures or features.

Overall, the present data does not support the indiscriminate use of standard or high-resolution CBCT imaging for endodontic evaluations prior to and after root canal treatment. Overall, CBCT should ideally be reserved for specific diagnostic questions such as complex clinical situations and complications especially when 2D imaging and/or the surgical microscope are deemed inadequate. The current results demonstrate that the acquisition mode is less significant for the subjective image quality of CBCT scans than the observer specialty, stage of treatment (including the type of post used), and type of tooth involved. Further research is needed to evaluate the potential of low-dose CBCT protocols for endodontics, especially as the effective dose for a CBCT with a small FOV and a low-dose mode could be as low as in the range of 1–2 panoramic views [12, 33, 34]. As there are many CBCT devices on the market, the present findings may at least partially be also dependent on device specifications, and thus cannot be directly extrapolated to other devices not tested in this study.

## Conclusions

From the findings of the present in vitro study, the following conclusions can be drawn:

- A routine use of standard or high-resolution mode settings for CBCT images for assessing root canal systems prior to or following root canal treatment should be questioned.
- Observer specialty, stage of treatment, type of post, and type of tooth were all factors influencing subjective image quality of CBCT scans.
- A low-dose CBCT mode for diagnostic purposes prior to or following root canal treatment results in favorable ratings regarding subjective image quality.
- As the data from the present study are based on an in vitro model distant from clinical situations, the findings need to be validated in future clinical investigations, ideally also using different CBCT devices.
